# Continuous Flow Photoelectrochemical Reactor with Gas Permeable Photocathode: Enhanced Photocurrent and Partial Current Density for CO_2_ Reduction

**DOI:** 10.1002/advs.202411348

**Published:** 2024-12-16

**Authors:** Hyunju Jung, Aqil Jamal, Issam Gereige, Tan Tien Nguyen, Joel W. Ager, Hee‐Tae Jung

**Affiliations:** ^1^ Department of Chemical and Biomolecular Engineering Korea Advanced Institute of Science and Technology (KAIST) 291 Daehak‐ro Yuseong‐gu Daejeon 34141 South Korea; ^2^ KAIST‐UC Berkeley‐Vietnam National University Climate Change Research Center KAIST 291 Daehak‐ro Yuseong‐gu Daejeon 34141 South Korea; ^3^ Saudi Aramco‐KAIST CO2 Management Center KAIST 291 Daehak‐ro Yuseong‐gu Daejeon 34141 South Korea; ^4^ Research and Development Center Saudi Aramco Dhahran 31311 Saudi Arabia; ^5^ National Key Lab for Digital Control and System Engineering Mechatronics Engineering Department VNU‐HCM‐Hochiminh City University of Technology 268 Ly Thuong Kiet Street, District 10 Ho Chi Minh City 72506 Viet Nam; ^6^ Chemical Sciences Division Lawrence Berkeley National Laboratory 1 Cyclotron Road Berkeley CA 94720 USA; ^7^ Department of Materials Science and Engineering University of California Berkeley CA 94720 USA

**Keywords:** continuous flow reactor, gas diffusion electrode, gas‐permeable photocathode, PEC catalyst, photoelectrochemical (PEC) CO_2_ reduction

## Abstract

Photoelectrochemical (PEC) CO_2_ reduction using a photocathode is an attractive method for making valuable chemical products due to its simplicity and lower overpotential requirements. However, previous PEC processes have often been diffusion‐limited leading to low production rates of the CO_2_ reduction reaction, due to inefficient gas diffusion through the liquid electrolyte to the catalyst surface, particularly at high current densities. In this study, a gas‐permeable photocathode in a continuous flow PEC reactor is incorporated, which facilitates the direct supply of CO_2_ gas to the photocathode‐electrolyte interface, unlike dark reaction‐based flow reactors. This concept is demonstrated using Ag‐TiO_2_ on carbon paper, illuminated through a quartz window and flowing liquid electrolyte. CO_2_ supply is managed via pressure and flow control on the non‐illuminated side of the carbon paper. The photocurrent density is significantly influenced by the flow rates and pressure of CO_2_ gas, and the electrolyte flow rates. Compared to the traditional H‐cell, the continuous PEC flow reactor achieves ≈10‐fold increase in CO faradaic efficiency, 30‐fold increase in production rate and 16‐fold increase in stability without catalyst modifications. This work provides essential insights into the design and application of continuous gas‐liquid flow PEC reactor systems, highlighting their potential for other PEC reactions.

## Graphical abstract

Continuous flow PEC reactor with a gas‐permeable Ag‐TiO₂ photocathode supplies CO₂ directly to the photocathode‐electrolyte interface. Controlled CO₂ and electrolyte flow rates, along with CO₂ pressure, enhance photocurrent density, achieving a 10x increase in CO faradaic efficiency, 30x production rate, and 16x stability improvement over traditional H‐cell systems. This design underscores the potential of continuous gas‐liquid PEC systems for efficient CO₂ reduction.

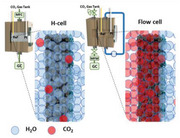

## 1 Introduction

Photoelectrochemical (PEC) reduction, which combines the strengths of electrochemical (EC) and photocatalytic (PC) reduction, is a promising solution for solar driven fuel production.^[^ ^1^, ^2^ ^]^ PEC systems can reduce complexity and decrease energy requirements due to their combined advantages, such as lower overpotentials compared to EC and acceleration of charge separation through bias‐induced band bending, leading to higher production rates than individual PC processes. These advantages make PEC a promising research area for enhancing the performance of a wide range of chemical reductions, including the CO_2_ reduction reaction (CO_2_RR)^[^ ^3^ ^]^ and the nitrogen reduction reaction (NRR).^[^ ^4^, ^5^ ^]^ However, conventional H‐cell PEC reactors, where reactants are supplied in liquid phases on the surface of the solid photocatalyst, have inherent limitations in terms of low production rates.

In the realm of electrochemical (EC) reduction of CO_2_ or N_2_, the development of flow reactor systems with an electrocatalytic gas diffusion electrode (GDE) has significantly enhanced the performance of various electrocatalytic reactions by increasing the mass transport rate in three‐phase continuous‐flow reactors.^[^ ^6^, ^7^, ^8^ ^]^ This GDE concept has also been adapted for light‐driven PEC systems, broadening the application of CO_2_ reduction technologies. In one approach, photovoltaic (PV) cells have been employed to power GDE‐equipped CO_2_ electrolysis devices, where light energy is converted into electricity that then powers the CO₂ reduction reaction.^[^ ^9^, ^10^, ^11^ ^]^ In another approach, GDE‐based cathodes for CO_2_RR have been coupled with photoanodes,^[^ ^12^, ^13^ ^]^ typically Si‐based, as counter electrodes.^[^ ^14^, ^15^ ^]^ These configurations allow direct conversion of gas‐phase CO₂, relying on external energy sources or light‐activated anodes. Our study advances these designs by combining light‐driven photocatalysis with gas‐phase CO_2_ reduction within a single continuous‐flow system. By introducing a gas‐permeable photocathode, we achieve both efficient CO_2_ transport and active light‐driven reactions at the electrode, addressing challenges seen in conventional PEC designs. This integration enhances mass transport and photocurrent generation, creating a stable triple‐phase interface ideal for selective and efficient CO_2_ reduction.

These studies raise the question as to whether CO_2_ mass transport limitations could be overcome at a photocathode. The use of a gas‐permeable “photo”‐electrode (PEC‐GDE) for this purpose becomes particularly intriguing in a three‐phase interface environment with sunlight participating directly in the photoreduction process. Electrons generated by light can participate directly in the desired reaction, like CO_2_RR, without transport losses. These considerations motivated us to develop a continuous‐flow reactor to improve the efficiency of PEC reactions using a gas‐permeable photocathode as the working electrode.

Our previous study has shown that PC CO_2_R can be done in a GDE geometry with greatly improved production rates and stability.^[^ ^16^ ^]^ In that work, we developed a continuous flow reactor in which the circulated liquid electrolyte and pressure‐controlled gaseous reactants flow on the surface of a solid photocatalyst. By employing CO_2_RR in the reactor, we observed a significant increase in the production rate ≈21 times compared to the batch reactor with 100+ hr stability. The mass transport on the photocatalyst surface improved due to the pressurized CO_2_ gas flow and the circulated water flow, accelerating desorption and preventing poisoning effects that deactivate photocatalyst activity.

In this study, we developed a gas permeable photocathode in a continuous‐flow PEC cell by combining the EC‐GDE^[^ ^6^, ^17^, ^18^ ^]^ and PC‐GDE^[^ ^16^ ^]^ approaches. The use of a gas‐permeable photocathode, wire‐type anode, and a reference electrode allowed the electrolyte and gas flow to influence the reaction conditions effectively. The concept was tested using photocathodes comprised of carbon paper and TiO_2_/Ag as a common form of photoelectrode.^[^ ^19^, ^20^ ^]^ Unlike H‐cell reactors, our continuous‐flow PEC‐GDE reactor offers an abundant and effective supply of gaseous reactants and proton donors in the electrolyte, resulting in a substantial increase in photoelectrochemical performance.

Several design constraints must be satisfied simultaneously in the PEC‐GDE concept. For the PEC function, it must be possible to illuminate a semiconductor (with co‐catalyst) material which must be electrically connected to the counter electrode (anode).^[^ ^21^, ^22^, ^23^ ^]^ For the GDE function, it must be possible to supply the gas‐phase reactant (CO_2_) and a source of protons (aqueous electrolyte) at the surface^[^ ^6^, ^17^ ^]^ of the semiconductor. While there are some exceptions,^[^ ^24^ ^]^ most typically employed GDE materials are opaque (e.g. carbon paper), steering us to use designs that would enable illumination through the liquid electrolyte.

## 2 Result and Discussion

### 2.1 Design and Configuration of the Continuous Flow PEC Reactor

**Figure 1:**
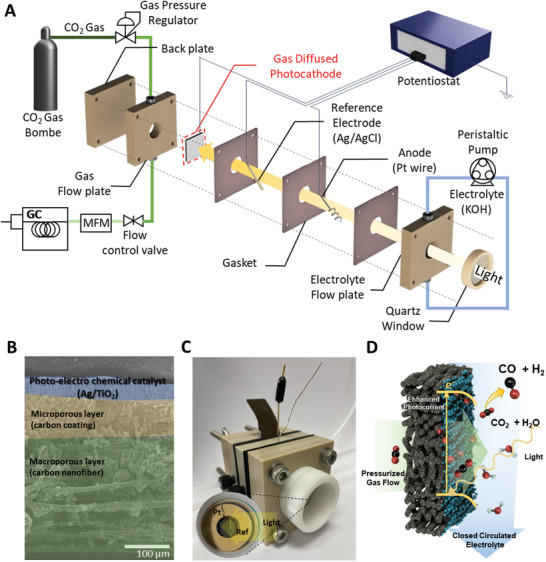
Reactor design and components of the photo‐electro chemical gas diffusion electrode (PEC‐GDE) reactor system. A) Magnified diagram of the continuous‐flow photoelectrocatalytic reactor and its configuration. B) Side image of the gas‐permeable photocathodeC) The photograph of reactor and minimized light‐blocking electrode arrangement in inserted image. in our system. D) PEC reaction scheme of the gas permeable photocathode in the continuous flow reactor.

**Figure** **1** illustrates the reactor components and system configuration we used to explore continuous‐flow PEC‐GDE reactor concepts. The reactor has a reactant gas flow plate, gaskets, an electrolyte flow plate, a quartz window plate, a Pt wire as anode, an Ag/AgCl electrode as reference electrode, and a gas permeable photocathode (Figure 1A, Figure S1, Supporting Information) which can be operated with continuous flow of both the CO_2_ reactant and the electrolyte. All of the reactor plates are composed of PEEK, which is inert to other chemicals and materials during the PEC reaction.

The gas‐permeable photocathode must have pores to allow gas diffusion, possess conductivity, and make good contact with the PEC catalyst, which is a semiconductor material. In addition, it must be hydrophobic to avoid flooding of the gas flow field by the liquid electrolyte. Following many rounds of experiments and optimization, we developed a simple and effective procedure. The gas permeable photocathode is fabricated through a spray‐coating process, where TiO₂, a PEC material, is deposited as a photocathode with Ag as the co‐catalyst onto a PTFE‐treated porous carbon gas diffusion electrode (Figure 1B and Figure S2, Supporting Information). This selection allows us to focus on comparing the performance of our PEC‐GDE reactor to an H‐cell reactor with the same PEC materials. However, it is noteworthy that our continuous flow PEC reactor is not limited to any specific reactions or GDE/PEC materials.

The porous layer structure of the carbon paper facilitates the unimpeded flow of CO_2_ gas reactants directly to the catalyst layer. An aqueous electrolyte solution is supplied to the catalyst layer, and its hydrophobic nature prevents excessive flow over the photocathode (Figure S3, Supporting Information). Through EDS mapping in Figure S4 (Supporting Information), it was confirmed that the hydrophobic treatment was evenly applied across the electrode surface.

To enable effective illumination of the TiO_2_/Ag, three key elements have been incorporated into the reactor: light direction, gas flow control, and electrolyte flow control. A pathway for light passage through the transparent electrolyte is constructed via a quartz window, reaching the gas‐permeable photocathode. Since the gas diffusion layer with conductive carbon is not transparent, light is directed toward the PEC catalyst side to initiate the photocatalytic reaction. Consequently, light passes through the reference and counter electrodes within the electrolyte to reach the photocathode. The use of a wire‐type PEEK leak‐free reference electrode and Pt counter electrode minimizes shading effects on the photocathode (Figure 1C). Also, they are positioned between low modulus rubber‐type gaskets. After assembling the cell, the gaskets envelop the electrode, allowing them to withstand the high pressure inside the reactor without leaks.

Precise control of gas and electrolyte flows and pressures are critical to maintaining the stability and efficiency of the PEC‐GDE system. Accurate regulation of these parameters ensures equilibrium between the reactant gas and electrolyte, thereby preventing adverse effects such as electrolyte overflows and gas bubbling into the electrolyte.^[^ ^16^ ^]^ To address this need, we implemented a continuous‐flow electrolyte system designed to closed‐circulate electrolytes between the gas‐permeable photocathode and a quartz window. This closed‐loop system facilitates uninterrupted electrolyte circulation, effectively mitigating temperature rise. On the opposing side of the electrolyte and photocathode, a CO_2_ gas flow system was established. This system is equipped with a gas pressure regulator, flow control valve, and mass flow meter, enabling precise control of both the pressure and flow rate of the CO_2_ gas. The CO_2_ is depressurized to the target pressure before entering the reactor, and the gas discharge is finely adjusted via a needle valve to maintain a stable pressure range of 1.1–1.4 bar. The mass flow meter provides real‐time monitoring, ensuring the accurate control of gas flow and pressure throughout the process.

Therefore, through all these processes, we expect to enhance the production rate for CO_2_ conversion in our flow reactor compared to the H‐cell, due to the direct utilization of gaseous reactants and liquid electrolyte by the photocathode (Figure 1D). Additionally, the use of precisely tuned flow media could create a favorable photocatalytic environment that maximizes photocurrent density, aiding in CO partial current enhancement.^[^ ^25^ ^]^

### 2.2 Impact of Flow Media on Photocurrent Density

**Figure 2:**
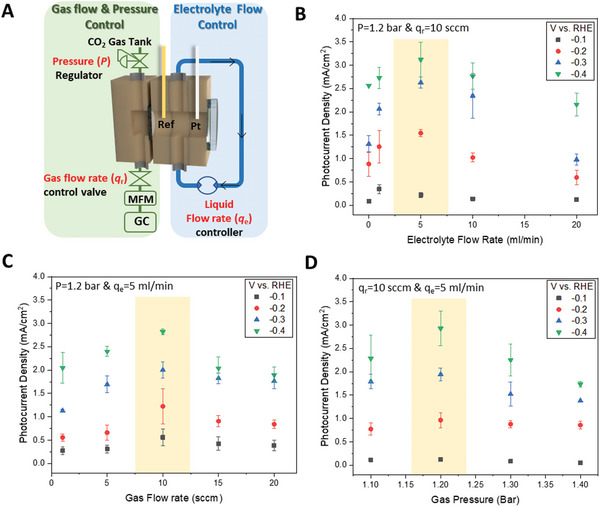
Effects of flow media on the photocurrent in flow‐type reactor system. A) illustration depicting the independent control of gas and electrolyte flow characteristics. Effect of B) cycled electrolyte flow rate, C) applied CO_2_ gas flow rate and D) gas pressure on the photocurrent density. Shadings indicates the regions of maximal photocurrent density. Error bars in (B)–(D) are standard deviations from 3 replicate experiments. 300 mW cm^−2^ illumination with Xe lamp, 1 m KOH electrolyte.

To enhance the selectivity of the CO_2_RR, the experiments were conducted in a 1 m KOH electrolyte, thereby minimizing concurrent hydrogen evolution. Under the range of potentials employed, −0.1 to −0.4 vs. RHE, the carbon paper by itself has a relatively small current (<5 mA cm^−2^) and negligible photocurrent under the employed illumination conditions (Xe lamp, 300 mW cm^−2^). The TiO_2_/Ag loaded photocathode has a substantial dark current (≈20 mA cm^−2^) but also has a clearly observable photocurrent response. In the following discussion, we focus on the photocurrent response, observing that, as expected, it is strongly influenced by the flow rates and pressure of CO_2_ gas, as well as the electrolyte flow rates. As shown in **Figure** **2**A, our reactor allows for systematic variation and optimization of the CO_2_ pressure (*P*), CO_2_ reactant flow rate (*q*_r_), and electrolyte flow rate (*q*_e_). Photocurrent values were determined by averaging at least three identical experiments, and all PEC reactions were conducted at room temperature.

Figure 2B illustrates the relationship between photocurrent and electrolyte flow rate at the optimal reactant pressure and flow rate (*P* ≈ 1.2 bar and *q*_r_ ≈ 10 sccm). As the electrolyte flow rate increases to ≈5 mL min^−1^, the photocurrent density also increases. However, with further increases in the electrolyte flow rate, the photocurrent density decreases across most potential ranges. This pattern is consistent for each potential condition, with the maximum photocurrent of ≈3 mA cm^−^^2^ at −0.4 V vs. RHE. Additionally, as the potential becomes more negative, the absolute magnitude of the photocurrent also increases correspondingly.

Figure 2C illustrates notable trends in photocurrent as a function of the CO_2_ gas flow rate with all other operational conditions remaining constant (*P* = 1.2 bar and *q*_e_ = 5 mL min^−1^). The applied CO_2_ gas flow rate affects the photocurrent density within this system. As the gas flow rate increases, the photocurrent density increases up to ≈10 sccm. However, with further increases in the gas flow rate, the photocurrent density decreases across all potential ranges.

**Figure 3:**
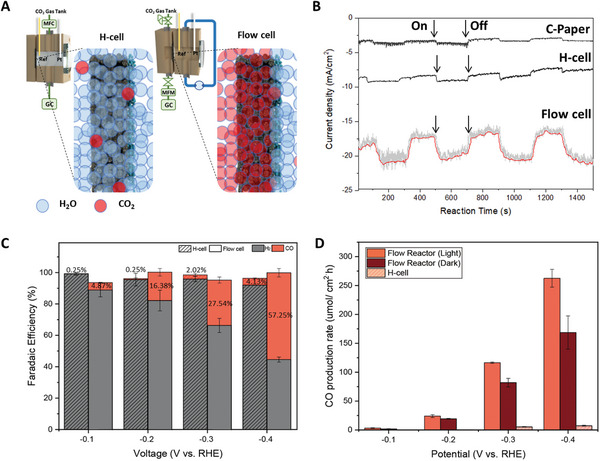
Comparison of the CO_2_ PEC reduction reaction performance of a gas diffusion photocathode, conducted in a continuous flow PEC reactor at *P* = 1.2, *q*_r_ = 10 sccm, and *q*_e_ = 5 ml min^−1^, with an H‐cell (*q*_r_ = 10 sccm) with an identically prepared carbon paper/TiO_2_/Ag cathode. A) Schematic images of H‐cell (left) and Flow cell (right). The blue circles represent H_2_O molecules, and the red circles represent CO_2_ molecules. B) Comparison of current density and photocurrent between Carbon paper (C‐paper), H‐cell, and flow cell at −0.4 V vs. RHE. Minor fluctuations in the measurements were due to the dynamic flow conditions in the electrolyte and gas system. C) Faradaic efficiency and D) CO production rates obtained over various potential ranges: −0.1, −0.2, −0.3, and −0.4 V vs. RHE with/without light irradiation. Error bars represent standard deviations from three replicate experiments. All experiments were conducted under following conditions: 300 mW cm^−^^2^ illumination using Xe lamp and 1 m KOH aqueous electrolyte.

Figure 2D demonstrates the impact of applied CO_2_ gas pressure on photocurrent. When varying only the reactant pressure while keeping other operational conditions constant (*q*_r_ = 10 sccm and *q*_e_ = 5 mL min^−1^), the photocurrent density exhibits a notable correlation with pressure changes. An increase in pressure initially boosts photocurrent density, peaking ≈1.2 bar. However, beyond this point, a further rise in pressure leads to a gradual decline in photocurrent. Clearly, the activity of the photocathode changes with variations in the reaction environment imposed by the reactor. To elucidate these effects, we conducted comparative experiments using an identically prepared carbon paper/TiO_2_/Ag GDEs in both an H‐cell‐type reactor and our continuous flow type PEC‐GDE cell. As shown in **Figure** **3**A, the diffusion coefficients and accessibility of reactants facing the electrodes vary depending on the reactor structure. For experiments with the H‐cell, *q*_r_ was set to 10 sccm at atmospheric pressure and room temperature. When performing experiments with PEC‐GDE test, they were conducted at room temperature, and the *q*_r_ was set at 10 sccm, *P* = 1.2 bar, and *q*_e_ = 5 mL min^−1^, where the photocurrent was maximally activated.

### 2.3 Comparison with H‐Cell: Faradaic Efficiency and Production Rate of CO₂ Reduction

We observed a significant improvement in current density and photocurrent magnitude in our continuous flow PEC reactor compared to the carbon paper (C‐paper) and the H‐cell (Figure 3B). In the H‐cell, after 200 s of light irradiation, current density increased but returned to its initial value once the light was turned off. At −0.4 V vs. RHE, the average current density of H‐cell was ≈−8 mA cm^−^^2^. The average photocurrent was ≈−1 mA cm^−2^. On the other hand, in the PEC‐GDE system, we observed an average current density of ≈−20 mA cm^−^^2^ and an average photocurrent density of −3 mA cm^−2^. The photocurrent of the C‐paper was negligible, ≈−0.3 mA cm^−^^2^, indicating an insignificant contribution to the total current density. This shows that a substantial portion of the observed current was due to a dark current originating from the PEC catalyst (TiO_2_/Ag).

Faradaic efficiency (FE) of PEC‐GDE was compared with H‐cell under different potential conditions (Figure 3C). HER was dominant in the H‐cell. As the applied potential increased, FE for CO showed a slight increase, although the proportion remained relatively small. However, using the same electrode composition, our PEC‐GDE significantly enhanced the FE for CO, with a maximum value of 57.25% at −0.4 V vs. RHE, ≈10x the value in the H‐cell at the same potential. At potentials more negative than −0.4 V, the CO Faradaic efficiency showed a further increasing trend, reaching ≈78% at −1.0 V (Figure S5).

The CO production rate in Figure 3D was calculated under three different reaction conditions: a flow reactor with light irradiation, a flow reactor without light irradiation, and an H‐cell with light irradiation. Across the entire potential range, the CO production rate in the continuous flow cell under light irradiation exhibited the highest value. However, the FE for CO in dark conditions (Figure S6, Supporting Information) was higher than that in light conditions. This is because, even with higher current due to maximized photocurrent under light irradiation, the HER also increased, resulting in a decrease in CO FE. However, the partial current for CO increased with light energy assistance (Figure S7 and S8, Supporting Information), and the reaction kinetics improved, as indicated by the lower Tafel slope in the flow cell compared to the H‐cell (Figure S9, Supporting Information). This enhancement led to an ≈1.3‐fold increase in the total CO production rate at −0.4 V vs. RHE in light conditions compared to dark conditions (Light: 262.6 µmol cm^−2^ h, Dark: 168.7 µmol cm^−2^ h). Furthermore, our system exhibited ≈33.3 times higher rates than the H‐cell (7.39 µmol cm^−^^2^ h).

### 2.4 Microenviornments Within the Reactor Based on Various Flow Media

**Figure 4:**
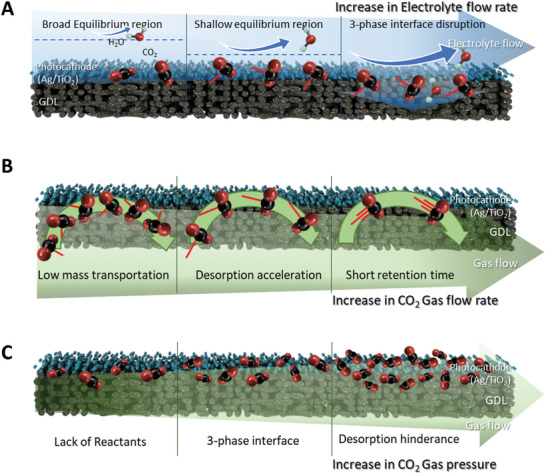
Illustration of gas diffused photocathode surface under various A) electrolyte flow rates, B) gas flow rates, and C) gas pressures. The red dashed lines connected to the CO_2_ molecules in (A) and (b) indicate the flow velocity of CO_2_ gas, providing a visual representation of the direction and relative speed of CO_2_ as it moves through the reactor under experimental conditions.

The increase in performance due to differences in reactors is related not only to CO_2_ accessibility through GDE^[^ ^26^ ^]^ but also to the carefully tuned microenvironment within the reactor, which is influenced by the media provided by the flow reactor. **Figure** **4** illustrates gas permeable photocathode by changing media flow rate and pressure. The flow of the electrolyte plays a vital role by effectively removing accumulated reaction products near the catalyst surface and promoting the diffusion of reactants (Figure 4A). At an electrolyte flow rate of 5 mL min^−1^, the balance between the hydraulic pressure generated by the electrolyte flow and the gas pressure at the catalyst surface optimally constructs the three‐phase interfaces at the catalyst layer. At flow rates below this threshold, the electrolyte flow fails to efficiently sweep away accumulated molecules near the catalyst layer, resulting in inefficient mass transport at the catalyst surface. Consequently, the rate of CO re‐adsorption increases, negatively affecting the overall reaction kinetics. Conversely, exceeding the optimal electrolyte flow rate increases hydraulic pressure, causing the electrolyte to progressively seep into the catalyst layer and impede mass transport. The electrolyte can even penetrate through the catalyst layer and move toward the gas layer. This dynamic interplay between flow rate and the formation of the three‐phase interface significantly influences the photocurrent response.

With the increase in gas flow rate, the mass transport of gas reactants on the catalyst surface increases (Figure 4B). The highest photocurrent density is observed at 10 sccm. This indicates that the reactant CO_2_ and the generated CO are efficiently adsorbed, reacted, and desorbed at this optimal flow rate. The residence time of the reactant is closely associated with the reaction time, adsorption time, and desorption time in the reactor. As the flow rate increases, the residence time of the reactants in the reactor decreases. Thus, at a residence time corresponding to ≈10 sccm, the photocatalytic reaction occurs most effectively. Above this rate, the residence time of CO_2_ molecules is insufficient for effective adsorption, photocatalytic reaction, and product desorption on the catalyst surface.

Gas pressure also plays a key role (Figure 4C). The highest photocurrent density is observed at 1.2 bar. Higher pressures decrease the photocurrent density. This decline could be attributed to the disruption of the triple‐phase interface due to excessive gas supply to the catalyst layer, affecting the balance between reactant and product adsorption‐desorption processes. This imbalance hinders efficient product desorption from the catalyst surface, thereby reducing overall photocurrent levels. Thus, maintaining an optimal gas pressure is crucial for sustaining an effective triple‐phase interface and optimizing the performance of the photocathode.

### 2.5 Stability of the Continuous Flow PEC Reactor with Gas‐Permeable Photocathode

**Figure 5:**
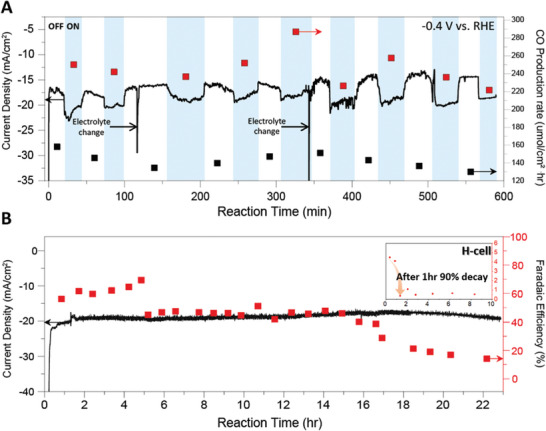
Stability tests of gas diffusion photocathode performed in continuous flow PEC reactor at −0.4 V vs. RHE in 1m KOH electrolyte, P = 1.2, *q*_r_ = 10 sccm, and *q*_e_ = 5 ml min^−1^. A) Current density and CO production rate with chopped 300 mW cm^−2^ light, irradiated and ceased intervalley ≈35 min for 580 min. Red and black squares are the CO production rates in the light and dark, respectively. B) Current density and Faradaic Efficiency with 100 mW cm^−2^ light without electrolyte changes. The inserted image shows the stability test (CO FE vs. time in h) conducted in the H‐cell.

In **Figure** **5**, we illustrate the excellent stability of the photocathode under a constant potential −0.4 V vs. RHE. Under consistent flow conditions as shown in Figure 3, when exposing the system to 300 mW cm^−^^2^ of chopped light at regular intervals for ≈10 h (Figure 5A), we observed variations in current density depending on the presence of light. This increase in photocurrent, reaching ≈3 mA cm^−^^2^, indicates that the photocurrent assists the overall current when the characteristics of the photocatalyst are in an optimized state. Calculating the production rate of the main product, CO, based on this increased photocurrent, we found that, on average, 249.0 ± 20.8 µmol cm^−2^ h of CO was produced in the presence of light, compared to an average of 143.8 ± 5.9 µmol cm^−2^ h in the absence of light.

Figure 5B shows sustained reaction stability under the illumination of 100 mW cm^−^^2^ for ≈23 h without any intervention. This demonstrated a sustained reaction with 71 ± 8% faradaic efficiency over ≈16 h. The abrupt decline in efficiency after 16 h is attributed to the accumulation of hydrophilic byproducts, which likely occurred due to the prolonged duration of the reaction. By measuring contact angle of initial state, during the reaction and after flooding of electrode, hydrophobicity remained until flooding (Figure S6, Supporting Information). On the other hand, the H‐cell type showed that the FE for CO dropped by about ten times after ≈1 h (Figure S7, Supporting Information). This high stability is attributed to the active reaction through flow characteristics and the introduction of a photocatalyst, even under vigorous light reactions.

## 3 Conclusion

In summary, we have developed a continuous flow PEC cell featuring a gas‐permeable photocathode for CO_2_ reduction (PEC‐GDE). By precisely controlling various parameters, including the flow rate and pressure of the reactant gas and the flow rate of the electrolyte, we can optimize the magnitude of the photogenerated current based on these flow characteristics. The reaction is most active when this current is maximized, which occurs with efficient adsorption and desorption of gas‐phase products and reactants varying with the media flow. This optimization has led to a substantial increase in the partial current density of CO production. When comparing the H‐cell reaction with the same materials under conditions where the photocatalyst material is most active, as indicated by photocurrent, we achieved a remarkable ≈30‐fold increase in the rate of CO production and a 10‐fold increase in CO FE. Additionally, the system maintained high stability for ∼16 h. When the electrolyte flow is appropriate, the desorption of reactants and products at the photocathode surface increases. With optimal CO_2_ flow rate and pressure, CO_2_ is abundantly supplied to the electrode surface, enhancing mass transfer. These findings suggest that our novel PEC cell design could potentially benefit not only CO₂ reduction reactions but also other photoelectrochemical processes and materials such as oxygen evolution reaction and nitrogen reduction reaction. This system's design and fluid dynamics insights can guide future PEC designs aimed at achieving high efficiency and selectivity, not only for CO production but also for other valuable reduction products, such as hydrocarbons and alcohols. We believe these advancements demonstrate the potential of our approach for sustainable energy and chemical production.

## Conflict of Interest

The authors declare no conflict of interest.

## Supporting information



Supporting Information

## Data Availability

The data that support the findings of this study are available in the supplementary material of this article.
