# Supplementary material for: Continuous Flow Photoelectrochemical Reactor with Gas Permeable Photocathode: Enhanced Photocurrent and Partial Current Density for CO2 Reduction
Source: Adv Sci (Weinh). 2024 Dec 16;12(6):2411348. doi: 10.1002/advs.202411348 (PMC11809356; doi:10.1002/advs.202411348)
Supplement: Supplementary file 1 — Supporting Information [file ADVS-12-2411348-s001.docx]

Supplementary Information

**Continuous Flow Photoelectrochemical Reactor with Gas Permeable Photocathode: Enhanced Photocurrent and Partial Current Density for CO_2_ Reduction**

Hyunju Jung^a,b,c,d^, Aqil Jamal^d^, Issam Gereige^d^, Tan Tien Nguyen ^b,e^, Joel W. Ager ^b,f,g^*, and Hee-Tae Jung^a,b,c,d^*.

AUTHOR ADDRESS

a Department of Chemical and Biomolecular Engineering, Korea Advanced Institute of Science and Technology (KAIST), 291 Daehak-ro, Yuseong-gu, Daejeon 34141, South Korea.

b KAIST-UC Berkeley-Vietnam National University Climate Change Research Center, KAIST, Daejeon 305-701, South Korea

c Saudi Aramco-KAIST CO_2_ Management Center, KAIST, Daejeon 305-701, South Korea.

d Saudi Aramco, Research and Development Center, Dhahran, 31311, Saudi Arabia

e National Key Lab for Digital Control and System Engineering, Mechatronics Engineering Department, VNU-HCM-Hochiminh City University of Technology, Vietnam Saudi Aramco-KAIST CO_2_ Management Center, KAIST, Daejeon 305-701, South Korea.

f Chemical Sciences Division, Lawrence Berkeley National Laboratory, 1 Cyclotron Road, Berkeley, California 94720, USA.

g Department of Materials Science and Engineering, University of California Berkeley, California 94720, USA

*Corresponding authors. Email: jwager@lbl.gov, heetae@kaist.ac.kr

**This file includes:**

Materials and Methods

Figs. S1 to S11

References 1

**Materials and Methods**

Materials

Methanol (MeOH), a Nafion 117 solution (~5 wt%), Polytetrafluoroethylene (PTFE) solution (60 wt%), n-hexane (> 95%) and TiO_2_ powder (P25) were purchased from Sigma-Aldrich. The carbon paper gas diffusion layer (GDL, Sigracet 39 BB) was purchased from Fuel Cell Store. All chemicals were used as received without further purification.

PTFE treatment of GDL

Initially, we subjected the GDL to a modified hydrophobization process^1^ involving the treatment with PTFE. To start, a 60 wt% PTFE emulsion was diluted with water, resulting in a 30 wt% PTFE emulsion. Subsequently, GDL was immersed in the 30 wt% PTFE emulsion for a duration of 10 seconds. Following this, the PTFE-soaked GDL was moved into hexane, and after a 10-second interval, it was transferred onto a dry acoustic sponge composed of polyurethane. Utilizing an airbrush connected to a N_2_ valve. The three-step process was repeated once, after which the GDL was placed in a ceramic boat and inserted into a tube furnace with Ar flow. The sintering program consisted of: (1) heating to 200 °C from room temperature at a ramp rate of 5 °C/min; (2) maintaining at 200 °C for 10 minutes; (3) heating to 320 °C from 200 °C at a ramp rate of 1 °C/min; (4) maintaining at 320 °C for 1 hour, followed by natural cooling to room temperature.

Fabrication of Gas Permeable Photocathode (PEC-GDE)

The catalyst ink was prepared by ultrasonically dispersing 100 mg of TiO_2_ powder with 200 µL of Nafion solution in 10 mL of MeOH for 30 minutes. Subsequently, the as-prepared catalyst ink was spray-coated onto a 25 cm^2^ GDL, and the electrode was left to dry overnight. Following the drying process, a 10 nm thick layer of Ag was deposited using an e-beam evaporator. The gas-permeable photocathode was then sliced into dimensions of 1.5 cm x 1.5 cm and connected using Cu tape.

Photochemical CO_2_ Reduction in H-cell

The H-cell tests for photoelectrochemical CO_2_ conversion performance were conducted in a custom cell made with PEEK. The photocathode was mounted on the back wall with a hole opening of 0.785 cm^2^. A platinum wire was used as the anode, and the reference electrode employed was 1M Ag/AgCl. Both compartments were filled with 15 ml of 1M KOH solution, and the electrolyte was continuously bubbled with CO_2_ at 10 sccm throughout the measurement. The light source used was a 300 W Xenon lamp, with a light intensity of 300 mW/cm^2^ in the reactor. The amounts of CO and CH_4_ obtained were analyzed using a gas chromatograph (GC; Agilent 7890GC) connected to the reactor.

Photochemical CO_2_ reaction in continuous flow reactor

All measurements ware carried out in a custom-made continuous flow reactor made with PEEK shown in Figure 1. The was built in-house and consists of a quartz window housing, gaskets, a catalyst layer, a gas flow plate, an electrolyte flow plate, and a back plate. The window housing, flow plates, and back plate were made from PEEK, owing to its chemical inertness, with sizes of 6×6×1 cm^3^. Pressurized gas flow was controlled using a gas regulator and a mass flow meter, and transported to gas flow plate adjoining one side of the catalyst layer through a ⅟_8_ʺ diameter Teflon-lined tube. The electrolyte plate was filled with water cycled by a peristaltic pump and the electrolyte feed was delivered to the other side of the catalyst layer through a ⅟_8_ʺ diameter Teflon tube. Light from a 300 W Xe lamp passed through the window housing and the electrolyte to the catalyst layer surface. All gaskets and flow plates had a 0.785 cm^2^ hole in the middle. All parts were assembled and fastened with four bolts. The product generated by the continuous reaction under light irradiation entered the in-line GC system for analysis during each of the set reaction times. Before measurement, the baseline was checked by measuring pure CO_2_ gas to detect any residual gas in the reactors.

Electrochemical Measurements

CO_2_RR was performed with chrono-amperometry (CA) measurementat each fixed potential for 30 min. During constant potential with iRcorrection, the gas products were detected by gas chromatography (Agilent 7890 GC), which was connected to the reactor. The potential was converted to RHE using the following equation:

E_RHE_ = E_AgCl_ + 0.059 pH + 0.209 V (1)

The FE for the products was calculated using the following equation:

FE (%) = nF x V * 100 j_Tot_^-1^ (2)

FE_CO_ is the calculated FE to CO in percentage

Characterization

The PEC-GDE was characterized with scanning electron microscopy (SEM) (Hitachi SU8230). The electrodes were cut in half to characterize the cross section in the center area of the electrodes with SEM. After electrolysis, the cathode was rinsed with DI water and acetone, and thoroughly dried before characterization.


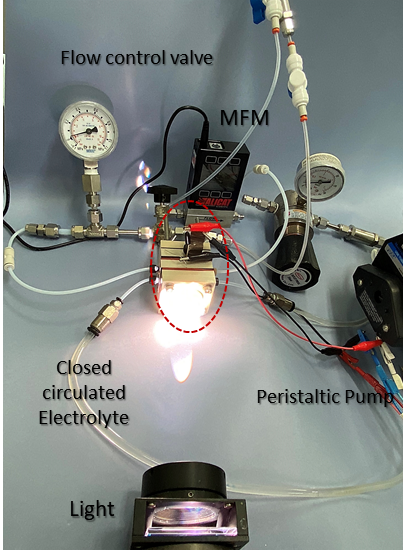


**Figure S1.** Photograph of the assembled continuous-flow photocatalytic reactor. Red circle shows the flow reactor


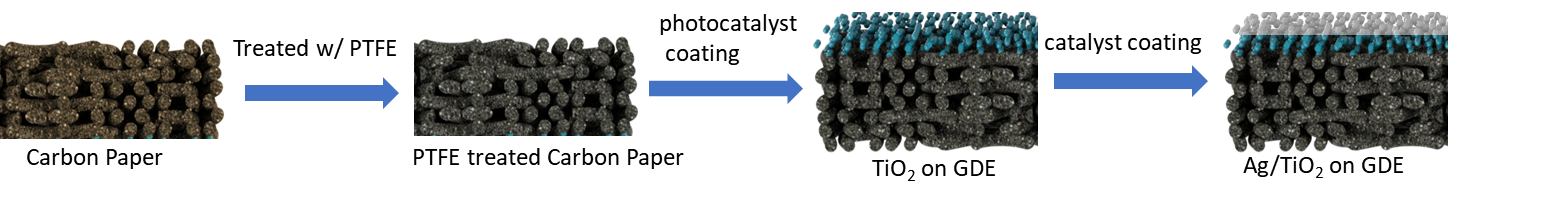


**Figure S2**. Fabrication process of gas permeable photocathode (PEC-GDE).


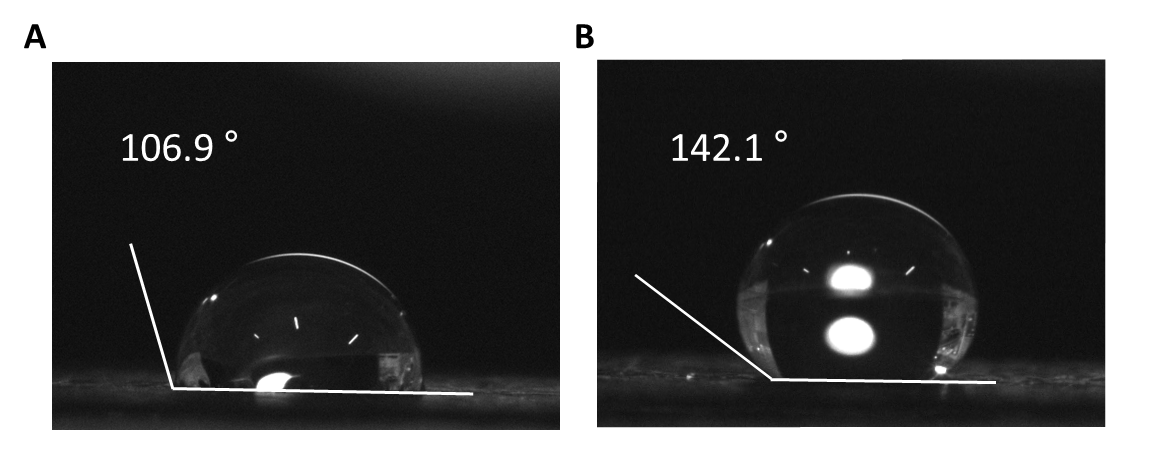


**Figure S3**. Contact angle of the carbon paper as gas diffused layer (A) before PTFE treatment, and (B) after PTFE treatment


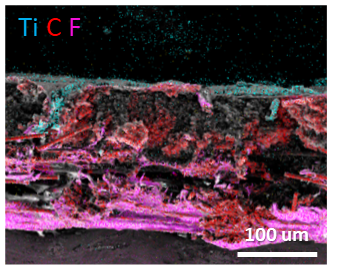


**Figure S4**. EDS mapping of elements Ti, C, F of the PEC-GDE.

**
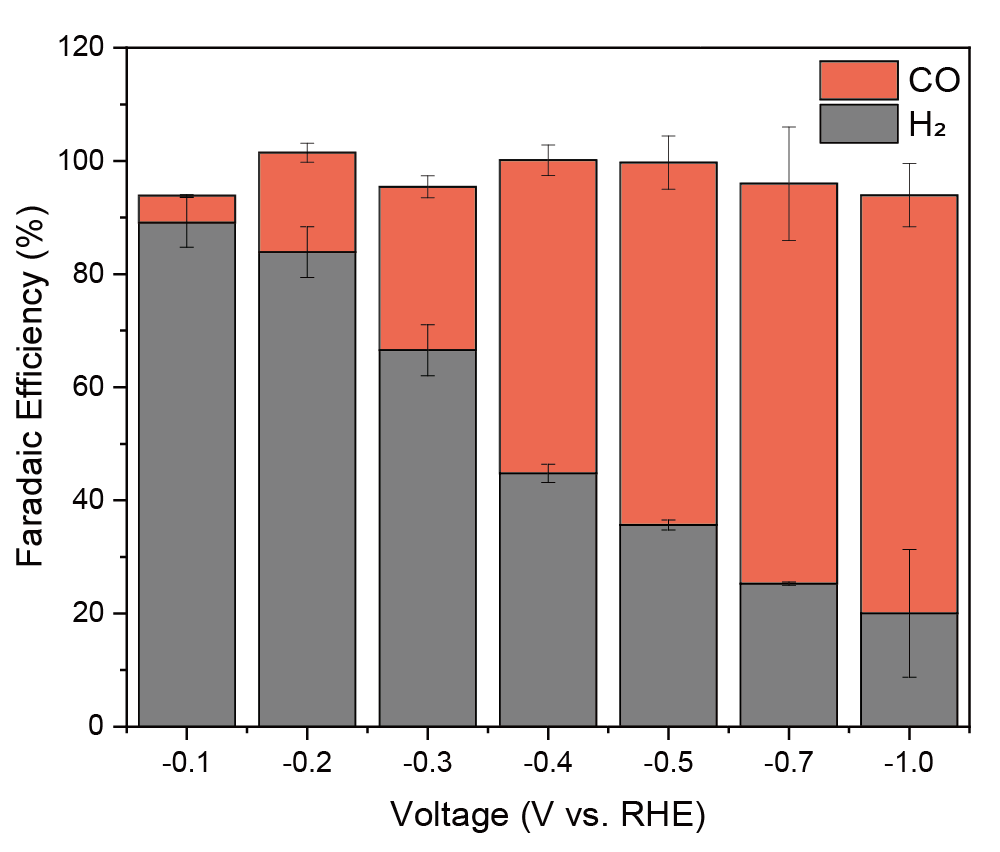
**

**Figure S5** Faradaic efficiency and CO production rates obtained over various potential ranges: -0.1, -0.2, -0.3, -0.4, -0.5, -0.7 and -1.0 V vs. RHE with light irradiation, conducted in a continuous flow PEC reactor at P = 1.2, qr = 10 sccm, and qe = 5 ml/min. Error bars represent standard deviations from three replicate experiments. Conditions: 300 mW/cm² illumination, 1 M KOH electrolyte.


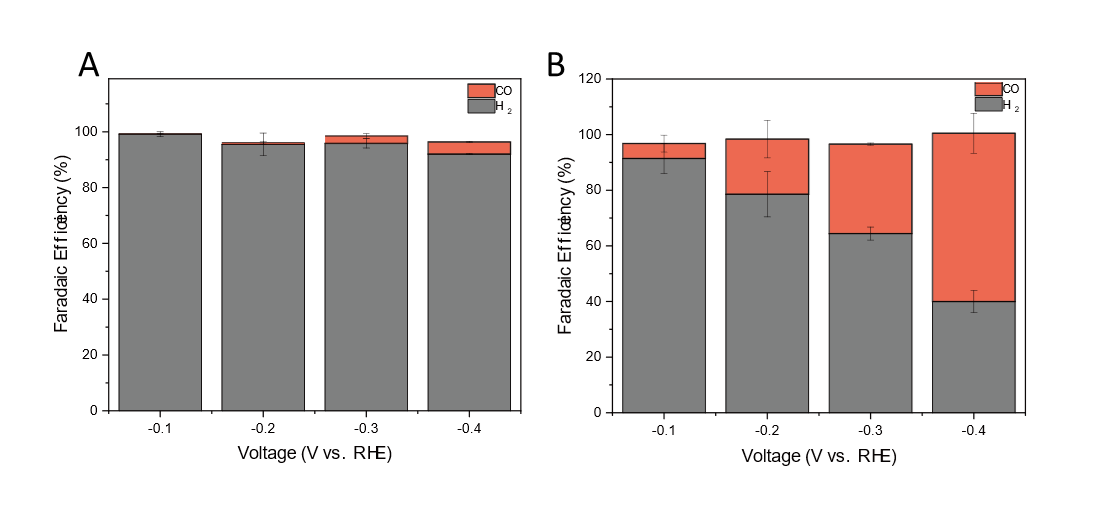


**Figure S6.** Faradaic efficiency at various potential range; -0.1, -0.2, -0.3 and - 0.4 V vs. RHE in (A) H-cell and (B) flow reactor without light irradiation. For flow reactor, Gas Pressure (*P*) = 1.2, Gas flow rate (*q*_r_) = 10 sccm, and electrolyte flow rate (*q*_e_) = 5 ml/min. For H-cell *q*_r_ = 10 sccm. Error bars are standard deviations from 3 replicate experiments. Conditions: 300 mW/cm² illumination with Xe lamp, 1 M KOH electrolyte.


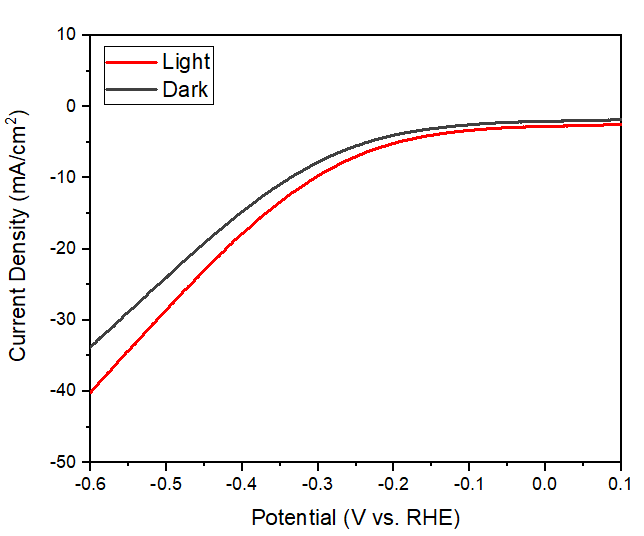


**Figure S7.** Current density (J) vs. voltage (V) plots of a carbon paper/TiO_2_/Ag PEC-GDE in 1 M KOH at scan rate 5 mV/s at the presence of light (red) and dark (black) in the continuous flow PEC reactor. P = 1.2, *q*_r_ = 10 sccm, and *q*_e_ = 5 ml/min. Illumination Conditions: 300 mW/cm² illumination with Xe lamp.


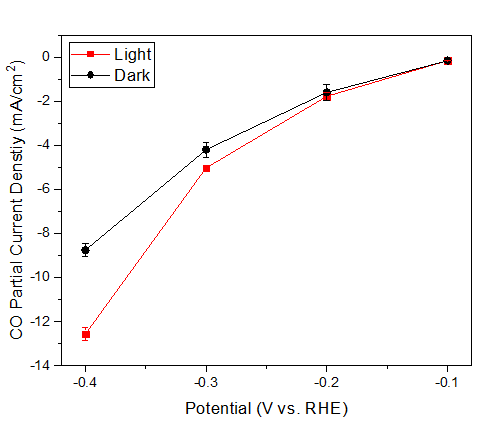


**Figure S8**. Partial Current Density of CO vs. voltage (V) plots of a carbon paper/TiO_2_/Ag PEC-GDE in 1 M KOH at the presence of light (red) and dark (black) in the continuous flow PEC reactor. P = 1.2, *q*_r_ = 10 sccm, and *q*_e_ = 5 ml/min. Illumination Conditions: 300 mW/cm² illumination with Xe lamp.

**
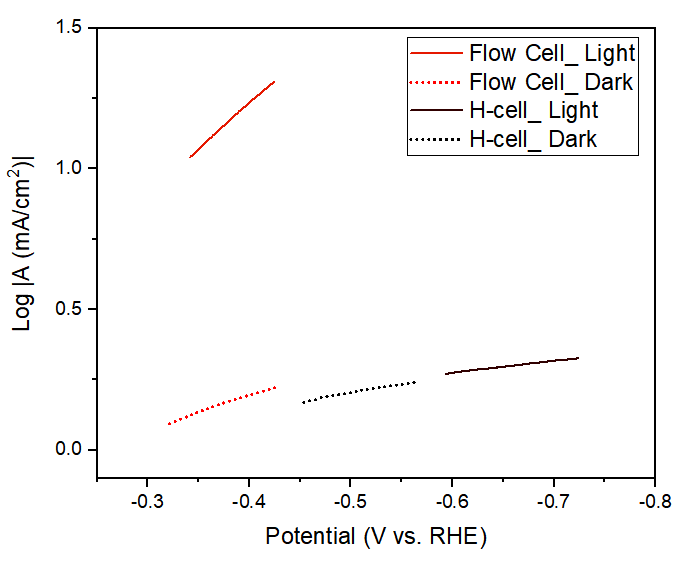
**

**Figure S9**. Tafel plots for the gas-permeable photocathode under light irradiation and dark conditions in both the flow cell and H-cell configurations.

**
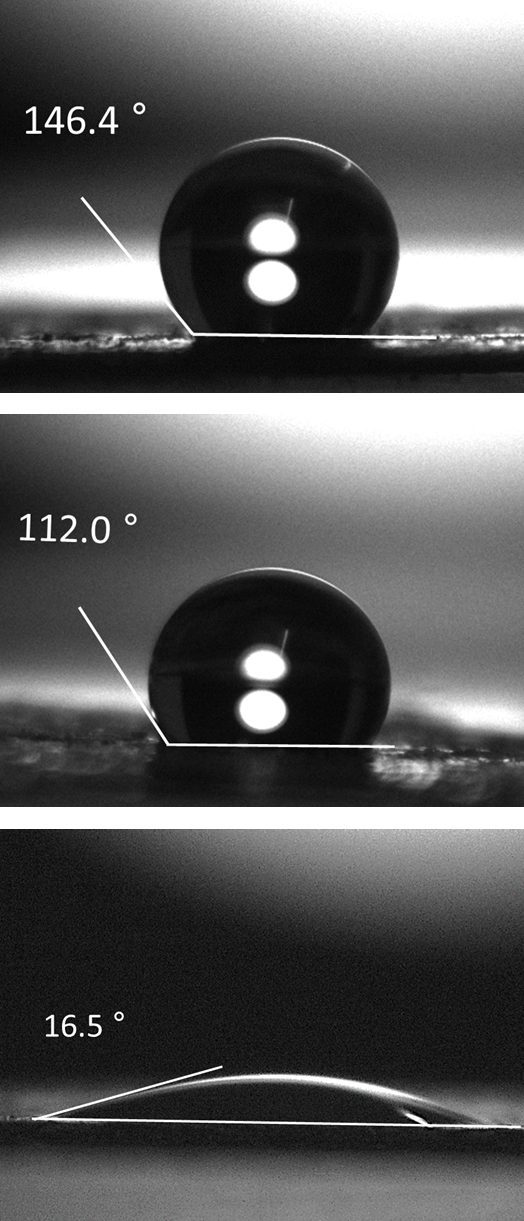
**

**Figure S10.** Contact angle of (A) the carbon paper/TiO_2_/Ag PEC-GDE cathode before reaction, (B) during reaction, and (C) after flooding.

**
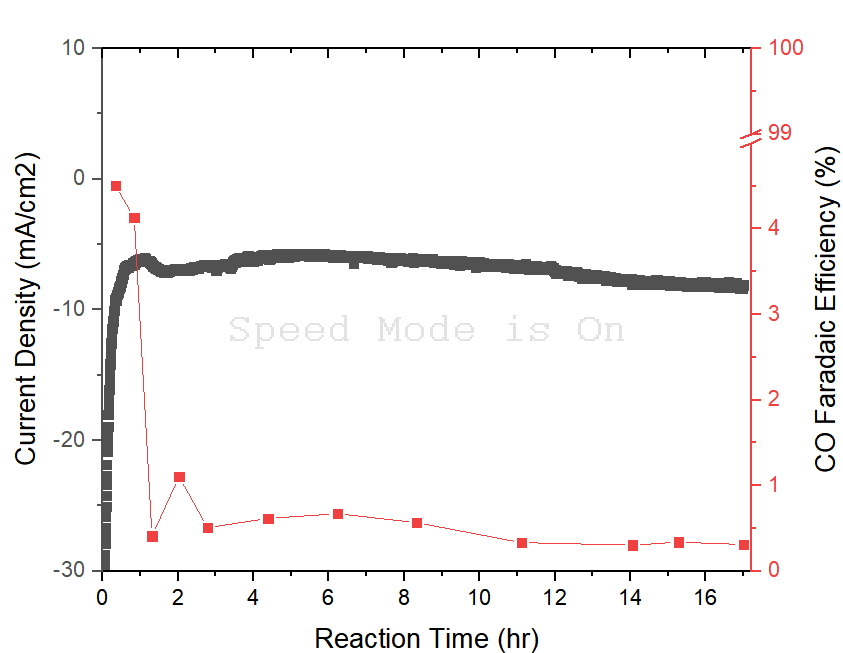
**

**Figure S11.** Stability tests of carbon paper/TiO_2_/Ag photocathode performed in H-cell type reactor at -0.4 V vs. RHE. Current density and CO Faradaic Efficiency with 100 mW/cm^2^ light with CO_2_ gas flow rate = 10 sccm.

**References and Notes**

**1** *L. Li, J. Chen, V. S. S. Mosali, Y. Liang, A. M. Bond, Q. Gu, J. Zhang, Angew. Chem. Int. Ed.* ***2022****, 61, e202208534*
